# The Role of Proteasomes in the Thymus

**DOI:** 10.3389/fimmu.2021.646209

**Published:** 2021-03-19

**Authors:** Melina Frantzeskakis, Yousuke Takahama, Izumi Ohigashi

**Affiliations:** ^1^Thymus Biology Section, Experimental Immunology Branch, National Cancer Institute, Bethesda, MD, United States; ^2^Division of Experimental Immunology, Institute of Advanced Medical Sciences, University of Tokushima, Tokushima, Japan

**Keywords:** thymus, TEC, thymoproteasome, immunoproteasome, thymic selection

## Abstract

The thymus provides a microenvironment that supports the generation and selection of T cells. Cortical thymic epithelial cells (cTECs) and medullary thymic epithelial cells (mTECs) are essential components of the thymic microenvironment and present MHC-associated self-antigens to developing thymocytes for the generation of immunocompetent and self-tolerant T cells. Proteasomes are multicomponent protease complexes that degrade ubiquitinated proteins and produce peptides that are destined to be associated with MHC class I molecules. cTECs specifically express thymoproteasomes that are essential for optimal positive selection of CD8^+^ T cells, whereas mTECs, which contribute to the establishment of self-tolerance in T cells, express immunoproteasomes. Immunoproteasomes are also detectable in dendritic cells and developing thymocytes, additionally contributing to T cell development in the thymus. In this review, we summarize the functions of proteasomes expressed in the thymus, focusing on recent findings pertaining to the functions of the thymoproteasomes and the immunoproteasomes.

## Introduction

Proteasomes degrade ubiquitinated proteins into peptide fragments in the cytoplasm, and these fragments are transported to the endoplasmic reticulum (ER) by TAP transporter. In the ER, the peptides are trimmed by aminopeptidases, loaded onto major histocompatibility complex class I (MHC-I) molecules, and transported to the cell surface for antigen recognition by T cells. Peptide fragments loaded onto MHC-I molecules are typically 8–10 amino acids in length and contain hydrophobic or basic residues at the carboxyl terminus.

The proteasome is a 26S protein complex comprised of a 20S enzymatic core particle located between two 19S regulator particles. The 20s core particle is a multi-catalytic protease complex made up of 28 subunits arranged in a cylindrical structure. The subunits of the proteasome are arranged into four heteroheptameric rings: two outer rings comprised of α subunits α1–α7 and two inner rings consisting of β subunits β1–β7. Within the proteasome, three subunits β1, β2, and β5 are responsible for the proteolytic activity. The chymotrypsin-like activity mediated by the β5 subunit enables the production of peptides enriched with hydrophobic C-terminal residues for high-affinity association with the peptide-binding groove of MHC-I molecules (1–3).

**Figure 1:**
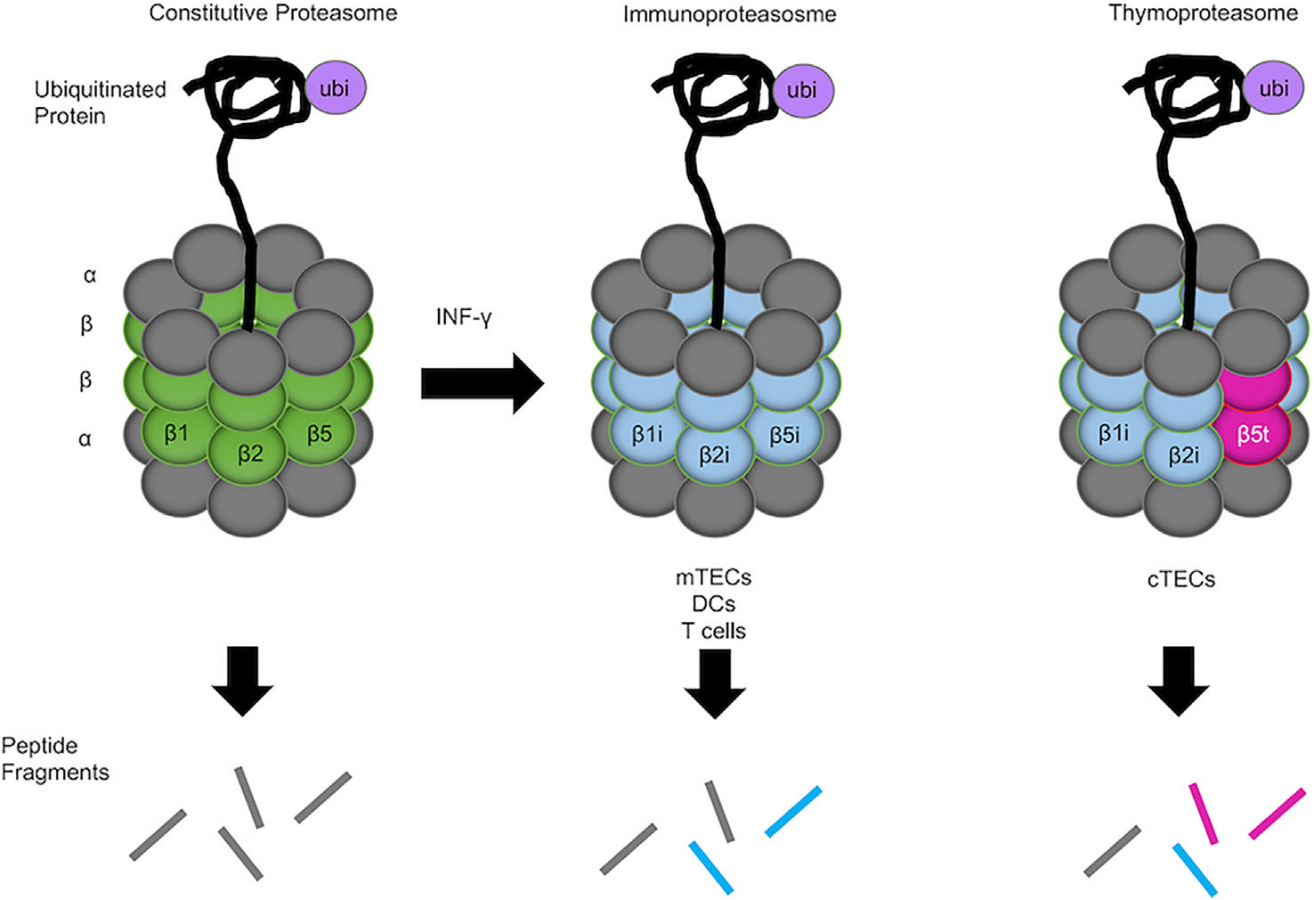
Constitutive proteasome, immunoproteasome, and thymoproteasome. 20s enzymatic cores have altered proteolytic activity and generated unique peptide fragments. The constitutive proteasome is ubiquitously expressed in the body and contains β1, β2, and β5 catalytic subunits (left). mTECs, DCs, and T cells in the thymus express the immunoproteasome, in which β1i, β2i, and β5i catalytic subunits are preferentially incorporated into the 20S core proteasome in lieu of β1, β2, and β5 subunits (middle). In cTECs in the thymus, a unique catalytic subunit β5t is specifically expressed instead of β5 or β5i and incorporated into the 20S core proteasome together with β1i and β2i, forming the thymoproteasome (right). Different catalytic subunits in different proteasomes provide different endopeptidase activity that alters the degraded peptides generated by the proteasomes (represented by different colored lines).

The proteolytic subunits of the proteasomes are diverse and vary among the constitutive proteasome (β1, β2, and β5), the immunoproteasome (β1i, β2i, and β5i), and the thymoproteasome (β1i, β2i, and β5t). These different catalytic subunits provide unique endopeptidase activity that alters the repertoire of degraded peptides generated by each proteasome (**Figure 1**). The immunoproteasome is abundant in interferon-γ-stimulated cells and constitutively expressed in various hematopoietic cells, including dendritic cells (DCs) and developing thymocytes (4). In the thymic medulla, medullary thymic epithelial cells (mTECs), which contribute to the establishment of self-tolerance in T cells, also express the immunoproteasome (5). In contrast, the thymoproteasome is exclusively and constitutively expressed in the cortex of the thymus by cortical thymic epithelial cells (cTECs) and is essential for optimal positive selection of CD8^+^ T cells (6–8).

In this review, we will discuss the functions of proteasomes expressed in the thymus, focusing on recent findings pertaining to the functions of immunoproteasomes and thymoproteasomes.

## Thymoproteasome in The Thymus for Cd8 T Cell Generation

The thymoproteasome contains a specific component β5t encoded by *Psmb11*, which is exclusively expressed by cTECs (6). Recent studies have identified that the cTEC-specific expression of β5t is directly regulated by Foxn1 (9, 10), a transcription factor important for the development of the thymic epithelium (11). It has been reported that β5t has altered proteolytic activity that leads to the preferential cleavage of proteins at hydrophilic peptide residues, reduced chymotrypsin-like activity, and reduced enzyme kinetics compared with β5, which probably result in the generation of a unique set of TCR ligands bound to MHC-I molecules on cTECs (6, 12). The β5t-containing proteasome produces peptides with not only different amino acid residues but also different quantities from those generated by other proteasomes, and the combination of these quantitative and qualitative differences may lead to the presentation of unique, or “private”, MHC-I-associated peptides (13). The inability to form peptide-MHC-I complexes with certain peptides may contribute to differential peptide display in the cortical and medullary regions of the thymus.

An *in vivo* function of the thymoproteasome was shown by analyses of β5t-deficient mice. β5t-deficient mice exhibited the selective reduction of CD4^-^CD8^+^ (CD8SP) thymocytes to approximately 25% of the normal number, whereas the cellularity of CD4^+^CD8^-^ (CD4SP) thymocytes was unaffected in these mice (6). The reduction of CD8SP thymocytes in β5t-deficient mice was primarily due to the decrease of CD69^+^TCRβ^+^ CD4^+^CD8^+^ (DP) thymocytes, which are post-selection cortical thymocytes, whereas the loss of pro-apoptotic protein Bim, which is required for the negative selection of T cells, increased CD69^+^TCRβ^+^ DP thymocytes in β5t-deficient thymus similar to that in β5t-sufficient thymus (8). These findings highlight the specific role of the thymoproteasome in the positive selection of CD8^+^ T cells, rather than the contribution of negative selection in the thymoproteasome-dependent generation of CD8SP thymocytes. In addition to being reduced in cell number, CD8^+^ T cells generated in β5t-deficient mice were altered in TCR repertoire, such as the differential usage of TCRVβ and TCRVα in CD8^+^ T cells but not in CD4^+^ T cells, and different TCR-transgenic CD8^+^ T cells were differentially susceptible to the loss of β5t in the thymus (7). Furthermore, CD8^+^ T cells generated in β5t-deficient thymus exhibited aberrant TCR responsiveness and immune response to infection (14). Therefore, these findings indicate that the thymoproteasome is required for the optimal positive selection of CD8^+^ T cells with functional TCR repertoire and determines the antigen responsiveness of mature CD8^+^ T cells in the periphery.

In addition to the analyses of β5t-deficient mice, the role of the thymoproteasome restricted in cTECs was examined by the analysis of β5t-transgenic mice in β5i-deficient background (β5i^-/-^β5t-Tg) in which the thymoproteasome was aberrantly expressed in antigen-presenting cells, including mTECs and DCs. Unlike β5t-deficient mice, β5i^-/-^β5t-Tg mice exhibited unaffected cellularity of TCRβ^low^CD69^-/+^CD103^+^ DP thymocytes, which are enriched with CD8^+^ lineage cells after positive selection, due to the retained expression of β5t in cTECs (15). On the other hand, the population of TCRβ^high^CD69^+^CD103^+^ DP thymocytes, which include post-selected CD8^+^ lineage cells, was lost in β5i^-/-^β5t-Tg mice, possibly resulting from enhanced negative selection (15). These findings are in agreement with the concept that a switch in self-peptides displayed by the cortex and the medulla may be important for the thymoproteasome-dependent development of CD8^+^ T cells in the thymus (16). Furthermore, the extra-thymic expression of β5t induced autoreactive responsiveness of CD8^+^ T cells that underwent thymoproteasome-dependent positive selection in the thymus (15). Therefore, cTEC-specific thymoproteasomes are important not only for the optimal generation of CD8^+^ T cells in the thymus but also for the prevention of aberrant T cell responses in the periphery. However, the systemic β5t-Tg mice were metabolically aberrant and showed weight loss due to the systemic reduction of chymotrypsin-like proteasomal activity (17). The study also showed that MHC-I expression was reduced in those β5i^-/-^β5t-Tg mice (15). Thus, the aberrant thymocyte development in their β5i^-/-^β5t-Tg mice may not be simply due to the loss of the MHC-I-associated peptide switching but possibly results from the combination of multiple abnormalities, including systemic aberrancy in metabolism, in those transgenic mice.

A recent study that employed RNA sequencing analysis identified that β5t regulated 850 cTEC-specific genes and exhibited a pervasive effect on CD4SP and CD8SP thymocyte development *via* the regulation of gene expression in cTECs (18). However, these results could not be reproduced in an additional study that found only minor differences in gene expression profiles between β5-sufficient cTECs and β5t-deficient cTECs through the combination of quantitative RT-PCR analysis and RNA sequencing analysis, and no significant effects on the development of CD4SP thymocytes (19). The contradictory results between these two studies may be due to differences in the genetic background of mice and the purity of isolated cTECs for transcriptomic analysis. It is also important to note that the former analysis lacked the verification of the modest differences in transcriptomic abundance detected in RNA sequencing analysis, by employing additional analysis, such as quantitative RT-PCR analysis. Furthermore, proteomic analysis revealed that β5t-deficient mice had reduced 20S core particle components (β1, β1i, β2, and β2i) except β5i and β5, which were elevated potentially to compensate for the loss of β5t, whereas neither chymotrypsin activity nor degradation of ubiquitinated proteins was apparently altered by the β5t deficiency (19). These results suggest that β5t may not pervasively regulate gene expression in cTECs, but is critical for maintaining normal proteasomal subunit composition in cTECs.

## Immunoproteasome in The Thymus for Antigen Processing and Beyond

The importance of the immunoproteasome in antigen presentation was examined by generating mice that completely lacked the immunoproteasome by the deletion of catalytic components: β1i-encoding *Psmb9*, β2i-encoding *Psmb10*, and β5i-encoding *Psmb8*. The defects detected in immunoproteasome-deficient mice resembled those detected in β1i-, β2i- or β5i-deficient mice. The number of CD8SP thymocytes in immunoproteasome-deficient mice decreased to approximately 50% of that in control mice (20), similar to the reduction detected in β1i-deficient mice (21). The expression of surface MHC-I molecules was reduced in the thymus and secondary lymphoid organs of immunoproteasome-deficient mice, similar to the reduced expression of surface MHC-I molecules in β5i-deficient mice (20, 22). The reduction of MHC-I expression was due to the reduced exportation of the peptide-MHC complex, not the instability of surface MHC-I molecules (20). Mass spectrometry analysis of peptides associated with MHC-I in splenocytes from immunoproteasome-deficient mice and control mice revealed that the MHC-I-associated peptide repertoire was altered by immunoproteasome deficiency (20). Altered generation of MHC-I-associated peptides in DCs was also detectable in β2i/β5i double-deficient mice (23). Although the repertoire of MHC-I-associated peptides in the thymus has not been examined, these results suggest that the generation of MHC-I-associated self-peptides in the thymus is also altered in immunoproteasome-deficient mice. As β1i and β2i are also components of the thymoproteasome, peptide generation in cTECs and thymic selection of cortical thymocytes may also be affected in immunoproteasome-deficient mice lacking β1i and/or β2i.

It has been reported that the number of polyclonal CD8SP thymocytes is not severely affected in β5i single-deficient mice (15, 22). However, one study disclosed that the generation of CD8^+^ T cells specific for viral glycoprotein GP_118-125_ was impaired in β5i-deficient mice, probably due to the altered thymic selection by the β5i deficiency in thymic antigen-presenting cells (24). Furthermore, the generation of naturally occurring self-peptide CPα1_92-99_, which likely contributes to the positive selection of ovalbumin (OVA)-specific OT-I T cells (25), is immunoproteasome-dependent, and β5i deficiency led to reduced generation of OT-I T cells in the thymus of OT-I-TCR transgenic mice (26). Therefore, β5i in the immunoproteasome within the thymus is involved in the generation of CD8^+^ T cells that express certain TCR specificities. It is possible that the deficiency in β1i more severely affects the generation of polyclonal CD8^+^ T cells (20, 21) than the deficiency in β5i (15, 22).

The role of immunoproteasomes beyond peptide processing in the thymus has been uncovered in several studies. mTECs exhibited promiscuous gene expression and synthesized more proteins than other thymic cells, which may require the alleviation of proteotoxic stress in these cells (27). The immunoproteasome deficiency caused by the genetic ablation of β2i-encoding *Psmb10* and β5i-encoding *Psmb8* in mice resulted in the selective reduction of mTEC cellularity and impaired mTEC regeneration due to the short half-lives of these cells and the exhaustion of their progenitors (27). Therefore, the immunoproteasome plays an important role in the homeostasis of mTECs.

An adoptive transfer of T cells isolated from mice deficient in immunoproteasome subunit β1i, β2i, or β5i exhibited decreased proliferation and increased apoptosis in virus-infected wild-type mice (28). Experiments in mixed bone marrow chimera mice showed that the altered homeostatic proliferation of T cells in secondary lymphoid organs was due to T cell-intrinsic deficiency in β2i (29). These studies suggest that the immunoproteasome is an intrinsic factor for the proliferation and survival of T cells, although the detailed mechanisms have yet to be elucidated. The immunoproteasome also regulates the activation of T cells. The specific inhibition of β5i reduced the production of IL-2 and INF-γ, as well as the expression of CD69 in T cells stimulated with anti-CD3 and anti-CD28 antibodies (30, 31). Furthermore, the phosphorylation of ERK, a molecule involved in the TCR signaling pathway, was reduced in activated T cells as a result of selective inhibition of β5i (31). Thus, the immunoproteasome plays a role in the regulation of TCR signaling.

Several studies that used non-immune cells have shown the involvement of the immunoproteasome in cellular senescence, aging, and longevity (32–34). Regarding cellular senescence, it has been reported that the TCR-mediated induction of the proteasome is impaired in senescence-associated PD-1^+^ CD44^high^ CD4^+^ T cells, and vice versa, the senescence phenotype is induced by proteasome inhibition (35). Although CD4^+^ T cells express both the constitutive proteasome and the immunoproteasome, these results indicate the involvement of proteasomes, including the immunoproteasome, in the senescence of T cells. However, whether the impaired induction of proteasomes by TCR stimulation is the cause or effect of T cell senescence remains unknown.

## The Thymus Lacking Immunoproteasomes and Thymoproteasomes

Three types of proteasomes are detected in the thymus: the constitutive proteasome, the immunoproteasome, and the thymoproteasome. Among major antigen-presenting cells in the thymus, cTECs in the cortex specifically express the thymoproteasome, whereas antigen-presenting cells in the medulla, such as mTECs and DCs, predominantly express the immunoproteasome. In one study, mice that were engineered to lack all components of those tissue-specific proteasomes were examined to address the function of the tissue-specific proteasomes in the thymus (16). In that study, mice deficient in β1i, β2i, β5i, and β5t (4KO mice) were generated. The mice were viable and bred normally, whereas their proteasomes were limited to constitutive proteasomes. Most strikingly, the 4KO mice had 90% fewer CD8^+^ T cells than control mice; this was substantially greater than the 75% reduction of CD8^+^ T cells seen in β5t-deficient mice (specifically deficient in thymoproteasomes) and the 50% reduction of CD8^+^ T cells seen in β1i/β2i/β5i-triple-deficient mice (deficient in immunoproteasomes) (16). There was no defect in the development of CD4^+^ T cells in the 4KO mice. Further evidence was provided to show that in the thymus of the 4KO mice, cortical thymocytes that undergo positive selection toward CD8^+^ T cells were detected. However, the 4KO mice had 68% fewer mature CD4^+^CD8^+^CD69^+^TCRβ^hi^CCR7^hi^ cells than wild-type mice, suggesting the occurrence of a developmental block downstream of positive selection. To assess the effect of tissue-specific proteasomes on negative selection, 4KO irradiated hosts were reconstituted with bone marrow from either wild-type or Bim-KO mice. Interestingly, the number of mature CD8^+^ thymocytes in the 4KO hosts reconstituted with Bim-KO bone marrow cells increased by 22.7-fold ± 8.8-fold than that in the 4KO hosts reconstituted with wild-type bone marrow cells. In contrast, the number of mature CD8^+^ thymocytes was only 5.0-fold ± 2.3-fold abundant in wild-type hosts reconstituted with Bim-KO bone marrow cells relative to that in wild-type hosts reconstituted with wild-type bone marrow cells. These data suggest that mature CD8^+^ thymocytes are lost due to increased events of apoptosis-dependent negative selection in the absence of tissue-specific proteasomes, which can be rescued by the loss of Bim (16). Based on these results, it was proposed that the restricted expression of tissue-specific proteasomes in the thymus, including cTEC-specific expression of the thymoproteasome and mTEC/DC-abundant expression of the immunoproteasome, facilitated a “peptide switch” between positive selection-inducing self-peptides presented by cTECs and negative selection-inducing self-peptides presented by mTECs and DCs. Such a switch in self-peptides may create a window for positively selected thymocytes to escape from subsequent negative selection. The difference in self-peptides displayed in the cortex and the medulla may be essential for the development of CD8+ T cells.

## Human Disease and Genetic Variations in Immunoproteasomes And Thymoproteasomes

The involvement of the immunoproteasome in human diseases has been reviewed by others (4, 36). It has been demonstrated that a single polymorphism in β1i or β5i is associated with risk of cancers and mutations in β5i lead to autoinflammatory syndromes. The alteration of immunoproteasome activity is also associated with the development of neurodegenerative diseases, such as Alzheimer disease and Huntington disease. Regarding the thymoproteasome, it has been shown that patients with Down syndrome have decreased expression of β5t, which may contribute to elevated mortality due to increased susceptibility to various cancers and infections (37). In addition, it has been shown that a single nucleotide polymorphism that changes the 49^th^ amino acid from glycine to serine (G49S) in the β5t protein is associated with Sjögren’s syndrome, an autoimmune disease that affects exocrine glands, specifically the lacrimal glands and the salivary glands (38). On the other hand, another study reported that the G49S variation had little association with severe human diseases, including cancer, hepatitis, and tuberculosis (39). The G49S variation impaired the post-translational processing of β5t protein in both mouse and human cells, whereas the homozygous G49S variation in mice reduced thymoproteasome expression in cTECs and decreased CD8^+^ T cell production in the thymus (39). Although the number of CD8^+^ T cells in homozygous human individuals has not been assessed, these findings suggest that G49S variation may affect the production of CD8^+^ T cells in the human thymus. A long-term and large-scale cohort study is expected to further deepen our understanding of the role of the thymoproteasome in human health.

## Conclusions and Perspectives

Since the discovery of the thymoproteasome by Murata, et al. in 2007, its functional significance and differential function from the immunoproteasome have been an interesting subject of studies. In the thymus, the processing of self-peptides that facilitate positive and negative selection events in developing thymocytes is presumably a major function of the thymoproteasome in cTECs and the immunoproteasome in mTECs and DCs. A study using fibroblasts with ectopic expression of the thymoproteasome and the immunoproteasome identified MHC-I-associated peptides generated by these proteasomes and showed a substantial difference in repertoire between thymoproteasome-dependent and immunoproteasome-dependent peptides (12). The difference in peptide generation between the thymoproteasome and the immunoproteasome was also shown in an experiment using a human lymphoblastoid cell line (13). Despite these findings, thymic selection-inducing self-peptides generated in thymic epithelium have not been identified yet. Therefore, biochemical analysis of MHC-I-associated peptides presented by cTECs and mTECs is required to improve our understanding of the difference between the thymoproteasome and the immunoproteasome, and contribute to the development of novel strategies to boost thymic selection for immunological disorders.

The explanation of the principle behind the differential expression of proteasomes between cortical and medullary thymic microenvironments remains controversial. As mentioned in this review, peptide switching between positive selection-inducing self-peptides presented by cTECs and negative selection-inducing self-peptides presented by mTECs and DCs may create a window to escape from the negative selection of positively selected thymocytes (15, 16). Alternatively, but not mutually exclusively, structural motifs in self-peptides generated by the thymoproteasome in cTECs may be advantageous for the optimal induction of positive selection. Indeed, we recently found that the thymoproteasome shapes the TCR repertoire directly in cortical thymocytes independent of the thymic medulla and independent of negative selection, indicating that the thymoproteasome hardwires the TCR repertoire of CD8^+^ T cells with cortical positive selection independent of negative selection (40). Additional studies are necessary to verify the nature of tissue-specific proteasomes in the thymus, which would ultimately improve current understanding of thymic selection in the thymus.

## Author Contributions

MF, YT, and IO wrote and edited the manuscript. All authors contributed to the article and approved the submitted version.

## Conflict of Interest

The authors declare that the research was conducted in the absence of any commercial or financial relationships that could be construed as a potential conflict of interest.

## References

[1] Coux O Tanaka K Goldberg AL. Structure and functions of the 20S and 26S proteasome. Annu Rev Biochem (1996) 65:801–47. 10.1146/annurev.bi.65.070196.004101 8811196

[2] Kloetzel P. Antigen processing by the proteasome. Nat Rev Mol Cell Biol (2001) 2:179–87. 10.1038/35056572 11265247

[3] Tanaka K. The proteasome: overview of structure and functions. Proc Jpn Acad Ser B Phys Biol Sci (2009) 85:12–36. 10.2183/pjab.85.12 PMC3524306 19145068

[4] Murata S Takahama Y Kasahara M Tanaka K. The immunoproteasome and thymoproteasome: functions, evolution and human disease. Nat Immunol (2018) 19:923–31. 10.1038/s41590-018-0186-z 30104634

[5] Groettrup M Kirk C Basler M. Proteasomes in immune cells: more than peptide producers? Nat Rev Immunol (2010) 10:73–8. 10.1038/nri2687 20010787

[6] Murata S Sasaki K Kishimoto T Niwa S Hayashi H Takahama Y. Regulation of CD8+ T cell development by thymus-specific proteasomes. Science (2007) 316:1337–53. 10.1126/science.1141915 17540904

[7] Nitta T Murata S Sasaki K Fujii H Mat Ripen A Ishimaru N. Thymoproteasome shapes immunocompetent repertoire of CD8+ T cells. Immunity (2010) 32:29–40. 10.1016/j.immuni.2009.10.009 20045355

[8] Xing Y Jameson S Hogquist K. Thymoproteasome subunit-β5t generates peptide-MHC complexes specialized for positive selection. PNAS (2013) 110:6979–84. 10.1073/pnas.1222244110 PMC3637736 23569244

[9] Žuklys S Handel A Zhanybekova S Govani F Keller M Maio S. Foxn1 regulates key target genes essential for T cell development in postnatal thymic epithelial cells. Nat Immunol (2016) 17:1206–17. 10.1038/ni.3537 PMC5033077 27548434

[10] Uddin M Ohigashi I Motosugi R Nakayama T Sakata M Hamazaki J. Foxn1-β5t transcriptional axis controls CD8+ T-cell production in the thymus. Nat Commun (2017) 8:14419. 10.1038/ncomms14419 28176764 PMC5309848

[11] Nehls M Kyewski B Messerle M Waldschutz R Schuddekopf K Smith AJH. Two genetically separable steps in the differentiation of thymic epithelium. Science (1996) 272:886–9. 10.1126/science.272.5263.88 8629026

[12] Sasaki K Takada K Ohte Y Kondo H Sorimachi H Tanaka K. Thymoproteasomes produce unique peptide motifs for positive selection of CD8+ T cells. Nat Commun (2015) 6:7484. 10.1038/ncomms8484 26099460 PMC4557289

[13] Kuckelkorn U Stübler S Textoris-Taube K Kilian C Niewienda A Henklein P. Proteolytic dynamics of human 20S thymoproteasome. J Biol Chem (2019) 294:7740–54. 10.1074/jbc.RA118.007347 PMC6514615 30914481

[14] Takada K Van Laethem F Xing Y Akane K Suzuki H Murata S. TCR affinity for thymoproteasome-dependent positively selecting peptides conditions antigen responsiveness in CD8+ T cells. Nat Immunol (2015) 16:1069–77. 10.1038/ni.3237 PMC4810782 26301566

[15] Tomaru U Konno S Miyajima S Kimoto R Onodera M Kiuchi S. Restricted expression of the thymoproteasome is required for thymic selection and peripheral homeostasis of CD8+ T cells. Cell Rep (2019) 26:639–51. 10.1016/j.celrep.2018.12.078 30650357

[16] Kincaid E Murata S Tanaka K Rock K. Specialized proteasome subunits have an essential role in the thymic selection of CD8+ T cells. Nat Immunol (2016) 17:938–46. 10.1038/ni.3480 PMC4955723 27294792

[17] Tomaru U Takahashi S Ishizu A Miyatake Y Gohda A Suzuki S. Decreased proteasomal activity causes age-related phenotypes and promotes the development of metabolic abnormalities. Am J Pathol (2012) 180:963–72. 10.1016/j.ajpath.2011.11.012 22210478

[18] Apavaloaei A Brochu S Dong M Rouette A Hardy M-P Villafano G. PSMB11 orchestrates the development of CD4 and CD8 thymocytes via regulation of gene expression in cortical thymic epithelial cells. J Immunol (2019) 202:966–78. 10.4049/jimmunol.1801288 30567730

[19] Ohigashi I Tanaka Y Kondo K Fujimori S Kondo H Palin A. Trans-omics impact of thymoproteasome in cortical thymic epithelial cells. Cell Rep (2019) 29:2901–16. 10.1016/j.celrep.2019.10.079 PMC6897492 31775054

[20] Kincaid E Che J York I Escobar H Reyes-Vargas E Delgado J. Mice completely lacking immunoproteasomes show major changes in antigen presentation. Nat Immunol (2012) 13:129–35. 10.1038/ni.2203 PMC3262888 22197977

[21] Van Kaer L Ashton-Rickardt P Eichelberger M Gaczynska M Nagashima K Rock K. Altered peptidase and viral-specific T cell response in LMP2 mutant mice. Immunity (1994) 1:533–41. 10.1016/1074-7613(94)90043-4 7600282

[22] Fehling H Swat W Laplace C Kühn R Raiewshy K Müller U. MHC class I expression in mice lacking the proteasome subunit LMP-7. Science (1994) 265:1234–7. 10.1126/science.8066463 8066463

[23] de Verteuil D Muratore-Schroeder T Granados D Fortier M-H Hardy M-P Bramoullé A. Deletion of immunoproteasome subunit imprints on the transcriptome and has a broad impact on peptides presented by major histocompatibility complex I molecules. Mol Cell Proteomics (2010) 9:2034–47. 10.1074/mcp.M900566-MCP200 PMC2938112 20484733

[24] Basler M Mundt S Groettrup M. The immunoproteasome subunit LMP7 is required in the murine thymus for filling up a hole in the T cell repertoire. Eur J Immunol (2018) 48:419–29. 10.1002/eji.201747282 29067678

[25] Hogquist KA Tomlinson AJ Kieper WC McGargill MA Hart MC Naylor S. Identification of a naturally occurring ligand for thymic positive selection. Immunity (1997) 6:389–99. 10.1016/s1074-7613(00)80282-4 9133418

[26] Osterloh P Linkemann K Tenzer S Rammensee H Radsak M Busch D. Proteasomes shape the repertoire of T cells participating in antigen-specific immune responses. PNAS (2006) 103:5042–7. 10.1073/pnas.0509256103 PMC1458791 16549793

[27] St-Pierre C Morgand E Benhammadi M Rouette A Hardy MP Gaboury L. Immunoproteasomes control the homeostasis of medullary thymic epithelial cells by alleviating proteotoxic stress. Cell Rep (2017) 21:2558–70. 10.1016/j.celrep.2017.10.121 29186691

[28] Moebius J van den Broek M Groettrup M Basler M. Immunoproteasomes are essential for survival and expansion of T cells in virus-infected mice. Eur J Immunol (2010) 40:3439–49. 10.1002/eji.201040620 21108466

[29] Zaiss D de Graaf N Sijts A. The proteasome immunosubunit multicatalytic endopeptidase complex-like 1 is a T-cell-intrinsic factor influencing homeostatic expansion. Infect Immun (2008) 76:1207–13. 10.1128/IAI.01134-07 PMC2258853 18160473

[30] Muchamuel T Basler M Aujay MA Suzuki E Kalim KW Lauer C. A selective inhibitor of the immunoproteasome subunit LMP7 blocks cytokine production and attenuates progression of experimental arthritis. Nat Med (2009) 15:781–7. 10.1038/nm.1978 19525961

[31] Schmidt C Berger T Groettrup M Basler M. Immunoproteasome inhibition impairs T and B cell activation by restraining ERK signaling and proteostasis. Front Immunol (2018) 9:2386. 10.3389/fimmu.2018.02386 30416500 PMC6212513

[32] Stratford FLL Chondrogianni N Trougakos IP Gonos ES Rivett AJ. Proteasome response to interferon-gamma is altered in senescent human fibroblasts. FEBS Lett (2006) 580:3989–94. 10.1016/j.febslet.2006.06.029 16806194

[33] Gavilán MP Castaño A Torres M Portavella M Caballero C Jiménez S. Age-related increase in the immunoproteasome content in rat hippocampus: molecular and functional aspects. J Neurochem (2009) 108:260–72. 10.1111/j.1471-4159.2008.05762.x 19012754

[34] Pickering AM Lehr M Miller RA. Lifespan of mice and primates correlates with immunoproteasome expression. J Clin Invest (2015) 125:2059–68. 10.1172/JCI80514 PMC4463211 25866968

[35] Arata Y Watanabe A Motosugi R Murakami R Goto T Hori S. Defective induction of the proteasome associated with T-cell receptor signaling underlies T-cell senescence. Genes Cells (2019) 24:801–13. 10.1111/gtc.12728 31621149

[36] Johnston-Carey HK Pomatto LCD Davies KJA. The immunoproteasome in oxidative stress, aging, and disease. Crit Rev Biochem Mol Biol (2015) 51:268–81. 10.3109/10409238.2016.1172554 PMC4968084 27098648

[37] Tomaru U Tsuji T Kiuchi S Ishizu A Suzuki A Otsuka N. Decreased expression of thymus-specific proteasome subunit β5t in down syndrome patients. Histopathology (2015) 67:235–44. 10.1111/his.12642 25556590

[38] Nitta T Kochi Y Muro R Tomofuji Y Okamura T Murata S. Human thymoproteasome variations influence CD8 T cell selection. Sci Immunol (2017) 2:eaan5165. 10.1126/sciimmunol.aan5165 28783658

[39] Ohigashi I Ohte Y Setoh K Nakase H Maekawa A Kiyonari H. A human PSMB11 variant affects thymoproteasome processing and CD8+ T cell production. JCI Insight (2017) 2:e93664. 10.1172/jci.insight.93664 PMC5436549 28515360

[40] Ohigashi I Frantzeskakis M Jacques A Fujimori S Ushio A Yamashita F. The thymoproteasome hardwires the TCR repertoire of CD8+ T cells in the cortex independent of negative selection. J Exp Med (2021) 218:e20201904. 10.1084/jem.20201904 33555295 PMC7873839

